# Reliability of operational data from pig herds and performance ratings by veterinarians and pig farmers collected during telephone interviews for the evaluation of a PCV2 piglet vaccination

**DOI:** 10.1186/s12917-014-0260-1

**Published:** 2014-10-28

**Authors:** Heiko Nathues, Johanna Meyer-Hamme, Petra Maass, Ruediger Goessl, Wibke Stansen, Rolf Steens, Elisabeth grosse Beilage

**Affiliations:** Field Station for Epidemiology, University of Veterinary Medicine Hannover, Foundation, Buescheler Street 9, Bakum, D-49456 Germany; Clinic for Swine, Department of Clinical Veterinary Medicine, Vetsuisse Faculty, University of Berne, Bremgarten Street 109a, Berne, CH-3012 Switzerland; Boehringer Ingelheim Animal Health GmbH, Binger Street 173, Ingelheim am Rhein, D-55216 Germany; Medical Data Service, Boehringer Ingelheim Pharma GmbH & Co KG, Binger Street 173, Ingelheim am Rhein, D-55216 Germany; Boehringer Ingelheim Vetmedica GmbH, Binger Street 173, Ingelheim am Rhein, D-55216 Germany

**Keywords:** Telephone interview, Survey, Porcine circovirus type 2, Ingelvac CircoFLEX®, Vaccination

## Abstract

**Background:**

The aim of the present study was to evaluate the feasibility of using a telephone survey in gaining an understanding of the possible herd and management factors influencing the performance (i.e. safety and efficacy) of a vaccine against porcine circovirus type 2 (PCV2) in a large number of herds and to estimate customers’ satisfaction.

**Results:**

Datasets from 227 pig herds that currently applied or have applied a PCV2 vaccine were analysed. Since 1-, 2- and 3-site production systems were surveyed, the herds were allocated in one of two subsets, where only applicable variables out of 180 were analysed. *Group 1* was comprised of herds with sows, suckling pigs and nursery pigs, whereas herds in *Group 2* in all cases kept fattening pigs. Overall 14 variables evaluating the subjective satisfaction with one particular PCV2 vaccine were comingled to an abstract dependent variable for further models, which was characterized by a binary outcome from a cluster analysis: good/excellent satisfaction (green cluster) and moderate satisfaction (red cluster). The other 166 variables comprised information about diagnostics, vaccination, housing, management, were considered as independent variables. In *Group 1*, herds using the vaccine due to recognised PCV2 related health problems (wasting, mortality or porcine dermatitis and nephropathy syndrome) had a 2.4-fold increased chance (1/OR) of belonging to the green cluster. In the final model for *Group 1*, the diagnosis of diseases other than PCV2, the reason for vaccine administration being other than PCV2-associated diseases and using a single injection of iron had significant influence on allocating into the green cluster (P < 0.05). In *Group 2*, only unchanged time or delay of time of vaccination influenced the satisfaction (P < 0.05).

**Conclusion:**

The methodology and statistical approach used in this study were feasible to scientifically assess “satisfaction”, and to determine factors influencing farmers’ and vets’ opinion about the safety and efficacy of a new vaccine.

**Electronic supplementary material:**

The online version of this article (doi:10.1186/s12917-014-0260-1) contains supplementary material, which is available to authorized users.

## Background

Porcine circovirus type 2 (PCV2) is associated with a number of disease syndromes and in recent years, ‘porcine circovirus associated diseases’ (PCVD) have been considered to have severe negative impact on global pig production [1]. Before availability of PCV2 vaccines, control of PCVD was focused on assuring good management and hygiene measures as well as reducing co-infections [2]. In 2008 the first PCV2 piglet vaccine was authorized in Europe. Safety and efficacy of that vaccine was confirmed in field studies for registration according to Good Clinical Practice [3,4]. In these negative controlled, blinded studies possible confounding factors are excluded to a large extent, allowing the detailed evaluation of vaccine safety (i.e. absence of adverse reactions, no significant negative impact on pig health, etc.) and efficacy under specific conditions. Vaccine efficacy is often determined by serological testing for specific antibodies, quantification of viral load in the serum and measuring production parameters such as daily weight gain and mortality. Due to practical reasons such controlled studies can only be performed in a limited number of herds which does not allow inclusion of the numerous factors that may have an impact on vaccine efficacy and safety in the field. The use of a telephone survey based on a subjective scaling system potentially allows the collection of data from a large number of herds representing a broad spectrum of typical conditions found in the field.

In the case of PCV2, the collection and evaluation of reliable and representative data from field trials on a larger scale is potentially complex since PCVD comprises different disease complexes [5] and can therefore have an impact on a large range of herd parameters. Since the reasons for applying a PCV2 vaccine can be varied (e.g. post weaning multi-systemic wasting syndrome, pneumonia, enteritis, etc.), the assessment of only one or two parameters may not be appropriate to sufficiently measure the vaccine efficacy. Furthermore, the collection of only a few selected herd parameters would not take into consideration the farmers’ and veterinarians’ subjective experiences with the vaccine, which might be a good predictor for safety and efficacy. In addition, the collection of reliable and standardised production data, as recorded in randomised and controlled clinical trials, may not be feasible when the vaccine is used in a large number of herds. In such cases ‘interviews’ seem to be a valuable tool to estimate safety and efficacy. When assessing individual and subjective experiences by means of interviews, investigators should take into account that the type of questioning can largely influence the validity and reliability of data [6]. Moreover, pre-tested questions for identical aspects should be used to improve the quality of information [7,8].

The aim of the present study was to evaluate to which extent a telephone poll is in general applicable to better understand the factors influencing the performance of a vaccine in a large number of herds and to estimate customers’ satisfaction (i.e. safety and efficacy of the vaccine).

## Methods

In February 2008, Ingelvac CircoFLEX® (Boehringer Ingelheim Vetmedica GmbH, Germany) received marketing authorization in the European Union. Since 2007, it was possible to use the product in Germany with a conditional license according to §17c Tierseuchengesetz (German law regulating notifiable diseases in animals and use of vaccines). The objective of this telephone survey based study was to collect and evaluate data from 2007 which could indicate ‘safety’ and ‘efficacy’, as well as to characterise factors influencing veterinarians’ and farmers’ satisfaction with Ingelvac CircoFLEX®.

### Development and pre-testing of the questionnaire

A standardised questionnaire (in German) was developed including 180 variables focussing on herd health, management, vaccination, husbandry, hygiene and on customer satisfaction (see Additional files 1 and 2). In order to receive consistently structured answers, 164 variables were assessed by closed questions (e.g. yes/no, scores from 1 = ’excellent’ to 6 = ’unsatisfactory’, etc.). Further experiences and occasional records were assessed by means of open questions (16 variables). For the purpose of validation, i.e. pre-testing, the principal investigator (JMH) contacted 10 farmers and their herd attending veterinarians, who had also used the PCV2 vaccine prior to authorization. They were all located in the South of Germany and their data was not included in the final analysis. During these exploratory telephone interviews, misunderstandings, unclear questions, missing variable levels, i.e. potential answers, were identified and the time needed for the individual interview, as well as the overall feasibility of the technique were evaluated. Subsequently, the questionnaire was revised according to the results of the validation process.

### Inclusion and exclusion criteria

According to the officially granted, farm-individual permissions in six German federal states covering the area of Northern Germany (Brandenburg, Mecklenburg-Vorpommern, Niedersachsen, Nordrhein-Westfalen, Sachsen-Anhalt and Schleswig-Holstein), the PCV2 vaccine had been used in 305 herds before the product was finally authorized. The contact details of these herds (n =305) and their attending veterinarians (n =133) were provided by the manufacturer of the vaccine in compliance with German data protection rules.

All these herds including their veterinarians were proposed to be included in the study and had been contacted by the principle investigator. If farmers or veterinarians refused voluntary participation, had not used the product although they had requested it or were unavailable due to other reasons (e.g. death, etc.), these herds were excluded from the study.

### Data collection

Practitioners were firstly contacted by post, explaining the aim and scope of the study, announcing that the interview will take place within the next seven days and indicating that the participation in the study is voluntary. Subsequently, veterinarians and the corresponding farmers were interviewed by the principal investigator. Each record was then entered into a digital form containing tick-boxes, drop-down menus and integer fields for coding and/or categorising given answers (Microsoft Office InfoPath 2003; www.microsoft.com). Macros and ‘if-then’ logics were used to check plausibility of the data entered within each filled form.

### Statistical analysis

In a first step, all variables assessed during the interviews were assigned to the group of dependent or to the group of independent variables. Overall 14 variables evaluating the subjective satisfaction with the vaccine were assigned to the group of Y-variables (dependent variables, Table 1). The variables IL_32 and IL_3132 are assessing the (perceived) current mortality among fattening pigs in the farm and the (perceived) reduction of mortality after introduction of the vaccination in the particular farm. These questions have been answered with absolute values instead of rating on a scale from 1 to 6 and, thus are answers are not shown in Table 1. The other 166 variables comprised by various information about diagnostics, vaccination, housing, management, were considered as X-variables (Table 2).

**Table 1:** **Y-Variables screened for feasibility of depicting the ‘outcome’**

**Code**	‘Subjective’ ratings* made by farmers and veterinarians (basis of the final independent variable)	**Mean**	**SD**
***Questions asked to the veterinarians***			
I_14	How would you estimate the overall health status of the pig herd *prior to the use* of the PCV2 vaccine?	3.71	1.01
I_15.1	How would you estimate the overall health status of the pig herd *after starting to use* the PCV2 vaccine (i.e. today!)?	2.08	0.64
I_17.1	How would you rate the handling of the vaccine?	1.96	0.40
I_18	How is the following sentence matching your opinion: “My positive expectations of the vaccine’s effect in the pig herd were fulfilled”	1.95	0.65
***Questions asked to the farmers***			
II_22	How would you estimate the compatibility of the PCV2 vaccine?	1.88	0.62
II_23	How would you estimate the overall health status of the pig herd *prior to the use* of the PCV2 vaccine?	3.83	1.08
II_24.1	How would you estimate the overall health status of the pig herd *after starting to use* the PCV2 vaccine (i.e. today!)?	2.16	0.68
II_27	How would you estimate the uniformity of growth in the nursery unit *prior to the use* of the PCV2 vaccine?	3.14	0.86
II_28	How would you estimate the uniformity of growth in the nursery unit *after starting to use* the PCV2 vaccine?	2.22	0.47
II_29	How would you estimate the uniformity of growth in the fattening unit *prior to the use* of the PCV2 vaccine?	3.74	0.65
II_30	How would you estimate the uniformity of growth in the fattening unit *after starting to use* the PCV2 vaccine?	2.23	0.52
II_33.1	How would you rate the handling of the vaccine?	2.05	0.39
II_34.1	How would you estimate the efficacy of the vaccine?	1.97	0.73
II_35	How is the following sentence matching your opinion: ‘My positive expectations of the vaccine’s effect in the pig herd were fulfilled’	2.01	0.90
***Calculated variables based on answers provided by veterinarians and farmers***			
D_1415	[I_15.1]-[I_14] ‘Change in the overall health status due to the use of a PCV2 vaccine’ (veterinarians’ suggestion)	1.63	0.95
D_2324	[II_24]-[II_23] ‘Change in the overall health status due to the use of a PCV2 vaccine’ (farmers’ suggestion)	1.65	1.06
D_2728	[II_28]-[II_27] ‘Change in the uniformity of growth in the nursery unit due to the use of a PCV2 vaccine’ (farmers’ suggestion)	0.92	0.92
D_2930	[II_30]-[II_29] ‘Change in the uniformity of growth in the fattening unit due to the use of a PCV2 vaccine’ (farmers’ suggestion)	1.51	0.83
D_3132	‘Change in the estimated mortality rate among fattening pigs due to the use of a PCV2 vaccine (farmers’ suggestion)	NA	NA

*All ratings were made on a scale ranging from ‘1’ = ‘excellent’ to ‘6’ = ‘unsatisfactory’/NA = not applicable.

**Table 2:** **Variables, which levels were assessed during telephone interviews with farmers and their veterinarians**

**Variable**	**Variable**
***General herd characteristics***	***Diagnosis of PCVD***
Production type	Clinical observations
Herd size	Post mortem inspections
Production rhythm	Laboratory examinations
Purchase of animals	Time between diagnosis and first vaccination
Hygiene measures	
	***Use of PCV2 vaccine***
***Production data***	Use of PCV2 vaccines in sows
Morbidity	Use of PCV2 vaccines in piglets
Mortality	Age of piglets when vaccinated
Growth rates	Switch in timing of vaccination
	Interruption of vaccination against PCV2
Sow & gilt management*	Use of antibiotics prior and after vaccination
Acclimatisation	Time between vaccination and other measures
Vaccination	
	***Experience with PCV2 vaccination***
Grower & finisher management*	Compatibility
Age when moving	Perceived efficacy
Vaccination	Influence of vaccination on pig health
Other treatments	Impact of vaccination on morbidity*
	Impact of vaccination on mortality*
Intention of continuous use of the PCV2 vaccine*	Impact of vaccination on growth rate*
	Overall satisfaction with the PCV2 vaccine

*Questions were asked to farmers only.

In this study 1-, 2- and 3-site production systems were surveyed. For the purpose of sound analysis, the herds were further investigated in two different subsets, in which only applicable variables were further tested. *Group 1* was comprised by 171 herds, which all housed nursery pigs (farrow-to-finish herds, piglet producing herds, wean-to-finish herds and specialised nursing herds). All 163 herds in *Group 2* kept fattening pigs (mandatory) and only optional other age and production groups (farrow-to-finish herds, wean-to-finish herds, fattening herds). Overall 107 herds were analysed in both subsets, because the kept nursery pigs (*Group 1*) and fattening pigs (*Group 2*). This concept of potentially using herds in both subsets is justified by the pigs’ age-dependent separation of factors that have been further analysed in each *Group*. Any potential bias due to this procedure will be discussed accordingly.

Due to the unexpected almost unanimous positive response, the statistical data analysis was done in a stepwise procedure:

1. Descriptive univariate analysis for X and Y variables of the raw dataset. This dataset included all variables of the questionnaire and all answers given by farmers of 227 appropriate herds and their veterinarians.
2. Descriptive univariate analysis for X and Y variables of the final dataset. In this dataset, herds with missing values in either variable were omitted because this resulted in conflicts with the tests used for further analysis (e.g. Chi-Square, regression analysis, etc.). The final analysis included 106 herds for *Group 1* and 99 herds for *Group 2*.

The subsequent analysis for the final dataset consists of the following steps:

- A principal components analysis (PCA) for the responses Y to analyse the dependencies among them. The examination was carried out separately for *Group 1* and *Group 2.* Principal components explaining similarity within this data were calculated and subsequently visualised using PROC%BIPLOT macro [9].
- A cluster analysis to construct distinct clusters of items showing similar scoring pattern for all responses. This approach was chosen, because a correlation analysis and modelling of X variables and individual responses or subsets is considered to yield no satisfactory results due to the low percentages of individual scores ≥4. Cluster analyses were performed for *Group 1* and *Group 2*, respectively. Herds were clustered according to the scoring given for each dependent variable (PROC FASTCLUS). Two sets of herds were guessed representing ‘high satisfaction’ (GREEN cluster) and ‘moderate satisfaction’ (RED cluster) among farmers and veterinarians. The outcome of this analysis was plotted in two dimensions obtained from the principal component analysis. Dependent variables were dropped from further data processing, when they were not adding discriminative power to the cluster analysis (i.e. they were equally expressed in both clusters).
- The resulting clusters formed with variables that had not been dropped (see above) were investigated for associations between the cluster variables and X-variables in order to identify which factors might influence customer satisfaction. These bivariate relations were analysed with Fisher’s exact test and the calculation of odds ratios (for 2 × 2 tables only) including confidence intervals (PROC FREQ), i.e. the comparison between GREEN and RED clusters. This procedure was run for corresponding variables of *Group 1* and *Group 2*. Variables were selected for further in-depth analysis, if the p-value of the Fisher-Exact test was <0.2, the minimum cell count in every cell was >5 and/or the association with the outcome variable was ‘biologically sound’. Moreover, independent variables were dropped when missing values were observed.
- A forward stepwise logistic regression model was fit to the relevant X variables considering main effects as well as two-way interactions. For the resulting final model the odds ratios of the included effects were estimated. The aim of the final analysis was to estimate the influence of the independent variables on the outcome considering potential two-way interactions among variables. Therefore, an automated stepwise logistic regression model was developed (PROC LOGISTIC, FORWARD (SLENTRY = 0.1), Option ODDSRATIO). The model was applied to data from *Group 1* and *Group 2*.

Data analysis was performed using the Statistical Analysis System (SAS®) for Windows version 8.2 and 9.2 (SAS® Institute Inc. Cary NC, USA).

This study was conducted in accordance with the research ethics requirements of the University of Veterinary Medicine Hannover. Due to the nature of the study and the low risk posed to participants, formal approval from the Ethics Committee was not a requirement at the time of the study. Potential participants were contacted by post with information explaining the purpose and nature of the study and inviting participation.

Participants were informed that their data would be kept anonymous and securely, and that any material potentially leading to identification would be removed. Subsequently, potential participants were contacted by telephone in order to provide further information. Participants were asked to provide verbal consent prior to the interview and it was made clear that by agreeing to be interviewed, they were agreeing to be part of the study.

## Results

Data from 243 herds were collected in this study, corresponding to a response rate of 79.7%. Reasons for non-response to this survey (n =62) were: (I) retirement, bankruptcy or death of the farmer or the veterinarian between the start of vaccination and the suggested time of the interview, (II) vaccine not used, although farmer and veterinarian had asked for a permission, (III) the herd attending veterinarian changed during the course of the evaluated time period, or (IV) the vaccine was not used for a sufficient time period (i.e. single use of the vaccine in only one batch of pigs).

Sixteen herds had to be excluded from the dataset, because either herds were selling all suckling pigs (n =4), were vaccinating only gilts during their acclimatisation period (n =4), their owner was not willing to participate in the telephone survey (n =5), or the farmer was not using the vaccine in question (n =3). In conclusion, in-depth analysis was performed based on data from 227 herds. *Group 1* was comprised of 171 herds (107 farrow-to-finish herds and 61 piglet producing herds and of 3 specialised nursery herds, all of them housing varied numbers of nursery pigs, whereas *Group 2* contained data of 163 herds, all raising grow-finish pigs (107 farrow-to-finish herds, 15 wean-to-finish herds and 41 fattening herds).

The level of ‘satisfaction’ with the PCV2 vaccine was consistently high in *Group 1* and *Group 2*. Particularly the efficiency, compatibility and handling of the vaccine received good to excellent evaluations by the majority of veterinarians and farmers, respectively. Due to this one-sided positive response sensitive multivariate tests (principal components analysis and cluster analysis) were used for further statistical analysis.

- Descriptive univariate analysis

Pre-selected independent X-variables and their levels are described in Table 3. For variables that assessed perceptions and remarks (16 open questions) a grouping of answers was not possible. Thus these 16 variables were omitted from the dataset. A further screening was conducted to identify variables associated with the ‘outcome’.

**Table 3:** **Independent X-variables assessed via telephone interview with veterinarians and farmers using a PCV2 vaccine in piglets prior to official release to the market**

**Variable**	**Level**	**Herds/level**	
		**(%)**	**(n)**
***General herd characteristics***			
Production type	Farrow to finish	47.1	107
	Farrow to nursery	26.9	61
	Only nursery	1.3	3
	Nursery and fattening	6.6	15
	Only fattening	18.1	41
Herd size/Sows^a^	≤500	78.0	131
	>500	20.8	35
	n.a.	1.2	2
Herd size/Fattening pigs ^b^	≤500	14.7	24
	501 - 2,000	60.7	99
	>2,000	21.5	35
	n.a.	3.1	5
***Sow management*** ^***a***^			
Acclimatisation of gilts^a^	Yes	61.7	103
	No	11.4	19
	Own re-breeding	21.0	35
	n.a.	6.0	10
Farrowing rhythm^a^	1-week	42.9	72
	2-week	20.8	35
	3-week	20.8	35
	Other	15.5	26
Storage of production data^a^	Electronic	89.3	150
	Hand written	10.1	17
	Nothing	0.6	1
AIAO in the farrowing units^a^	Yes	91.1	153
	No	8.9	15
Vaccination of sows [S] and gilts [G] against^a^	PRRSV [S]	77.8	130
	PRRSV [G]	79.6	133
	PCV2 [S]	13.2	22
	PCV2 [G]	26.4	44
	Swine influenza virus	33.5	56
	Porcine Parvovirus & *E. rhusiopathiae*	98.2	164
	*P. multocida*	4.8	8
	*E. coli*	24.0	40
	Autogenous vaccines	21.0	35
***Piglet management***			
Age of piglets at weaning (days)^a^	21	44.1	74
	22 – 42	54.8	92
	n.a.	1.2	2
Age of piglets when castrated^a^	≤7	95.8	161
	8 – 14	4.2	7
Frequency of injecting iron^a^	Once	67.9	114
	Twice	32.1	54
Vaccination of piglets against^c^	*M. hyopneumoniae*	71.9	133
	using a 1-shot vaccine	40.5	75
	using a 2-shot vaccine	19.5	36
	n.a.	18.4	34
	PRRSV	31.4	58
	*A. pleuropneumoniae*	1.6	3
	*L. intracellularis*	10.3	19
	Autogenous vaccines	1.6	3
***Management of fattening pigs***			
AIAO in the fattening units^b^	Yes (by barn)	8.6	14
	Yes (by compartment)	77.9	127
	No	13.5	22
Storage of production data^b^	Electronic	16.6	27
	Hand written	81.0	132
	No	2.5	4
Disposition of low performing pigs before restocking^b^	Moving to younger pigs	9.8	16
	Euthanasia	9.2	15
	Hospital compartment	25.8	42
	Hospital pen	33.1	54
	Original pen	9.8	16
	Others	12.3	20
***PCV2 vaccination issues***			
Indication to use the vaccine (farmers’ answer)	Stunted growth	59.9	136
	Respiratory diseases	26.0	59
	Enteritis	4.9	11
	PDNS	44.5	101
	Increased mortality	56.8	129
	Marketing	29.1	66
	Other reasons	33.5	72
Indication to use the vaccine (veterinarians’ answer	Stunted growth	59.5	135
	Respiratory diseases	37.0	84
	Enteritis	15.4	35
	PDNS	45.8	104
	Increased mortality	47.6	108
	Marketing	26.9	61
	Other reasons	26.0	59
Duration of symptoms according to the indication before the vaccine was used for the first time (month)	<3	12.3	28
	3 – 6	4.4	10
	>6	75.3	171
	n.a.	7.9	18
Diagnostics (other than clinical examination) before vaccination	Yes	79.7	181
	No	20.3	46
Type of diagnostics	Antibody detection by ELISA	16.3	37
	of these IgM - IgG ELISA	1.3	3
	PCR	56.8	129
	Necropsy (only gross lesions)	39.2	89
	Histology	0.0	0
	Immuno-histology	1.3	3
Pathogens other than PCV2 found during these examination	Respiratory pathogens excl. PRRSV	18.1	41
	PRRSV or PRRSV & others	13.7	31
	*Sc. suis* or *Sc. suis* & others	10.6	24
	*L. intracellularis* & others	9.7	22
	Enteric pathogens excl. *L. intracellularis*	4.0	9
	Others	0.4	1
	None	43.6	99
Herd health ranking prior to the use of the PCV2 vaccine (farmers’ answer)	1 (excellent)	0.0	0
	2	11.5	26
	3	22.5	51
	4	33.5	76
	5	19.4	44
	6 (unsatisfactory)	5.3	12
	k. A.	7.9	18
Herd health ranking prior to the use of the PCV2 vaccine (veterinarians’ answer)	1 (excellent)	0.0	0
	2	11.5	26
	3	25.1	57
	4	35.2	80
	5	15.9	36
	6 (unsatisfactory)	3.1	7
	k. A.	9.3	21
Time between first use of the PCV2 vaccine and the telephone interview (month)	1 – 6	13.2	30
	7 – 12	73.6	167
	13 – 18	11.0	25
	n.a.	2.2	5
Age of piglets when vaccinated against PCV2 (days)	≤14	28.6	65
	15 – 21	29.1	66
	22 – 28	11.5	26
	29 – 69	8.8	20
	≥ 70	11.9	27
	Unknown*	10.1	23
Stage of production, when pigs are vaccinated against PCV2	Suckling period	63.4	144
	Nursery period	13.7	31
	Fattening period	12.8	29
	Suckling or nursery period*	10.1	23
***Timing of vaccination***			
Time between PCV2_vaccination and 1^st^ (or 2^nd^) vaccination against *M. hyopneumoniae* (days) ^c^(farmers´ answer)	0 (in parallel)	8.7	16
	1 – 7	7.0	13
	8 – 14	4.4	10
	≥15	2.7	5
Time between PCV2_vaccination and 1^st^ (or 2^nd^) vaccination against *M. hyopneumoniae* (days) ^c^(veterinarians’ answer)	0 (in parallel)	22.2	41
	1 – 7	10.8	20
	8 – 14	22.7	42
	≥15	13.2	30
Time between PCV2_vaccination and PRRSV vaccination (days) ^c^ (farmers’ answer)	0 (in parallel)	8.7	16
	1 – 7	0.5	1
	8 – 14	1.1	2
	≥15	1.1	2
Time between PCV2_vaccination and PRRSV vaccination (days)^c^ (veterinarians’ answer)	0 (in parallel)	18.9	35
	1 – 7	6.5	12
	8 – 14	3.2	6
	≥15	2.7	5
Substances applied in parallel with the PCV2 vaccine on the other side of the neck	Nothing	53.3	121
	*M. hyopneumoniae* vaccine	24.7	56
	PRRSV vaccine	13.2	30
	*M. hyopneumoniae* & PRRSV vaccine	2.2	5
	Others**	6.6	15
Castration and PCV2 vaccination in parallel	No	99.6	226
	Yes	0.4	1
Interruption of the PCV2 vaccination	No	92.5	210
	Yes	7.5	17
Actual use of the PCV2 vaccine at the time of the interview	No	10.1	23
	Yes	89.9	204
Change in the timing of PCV2 vaccination since started	No	79.7	181
	Yes	14.1	32
	n.a.	0.9	2
Use of other PCV2 vaccines than Ingelvac CircoFLEX® in piglets	No	87.7	199
	Porcilis® PCV (MSD)	6.6	15
	Suvaxyn® PCV 2 (Pfizer)	4.9	11
	Circovac® (Merial)	0.9	2
Reasons for the use of other PCV2 vaccines in piglets	Out of stock	10.1	23
	Insufficient efficacy	0.4	1
	n.a.	1.8	4

^a^only herds housing sows (n =167).

^b^only herds housing fattening pigs (n =163).

^c^only herds housing suckling and nursery pigs (n =185).

n.a.: not answered.

*pigs were either vaccinated during their suckling period or nursery period; the exact point in time was unknown.

**injection of iron, antimicrobials or other vaccines.

b) Principal component analysis

In order to analyse the relations among the responses, a principal components analysis was done for the Y variables:

- Group 1:
  I_15.1, D_1415, I_18, II_24.1, D_2324, II_28, D_2728 , II_34.1, II_35

- Group 2:
  I_15.1, D_1415, I_18, II_24.1, D_2324, II_30, D_2930, II_32, D_3132, II_34.1, II_35

**Figure 1:**
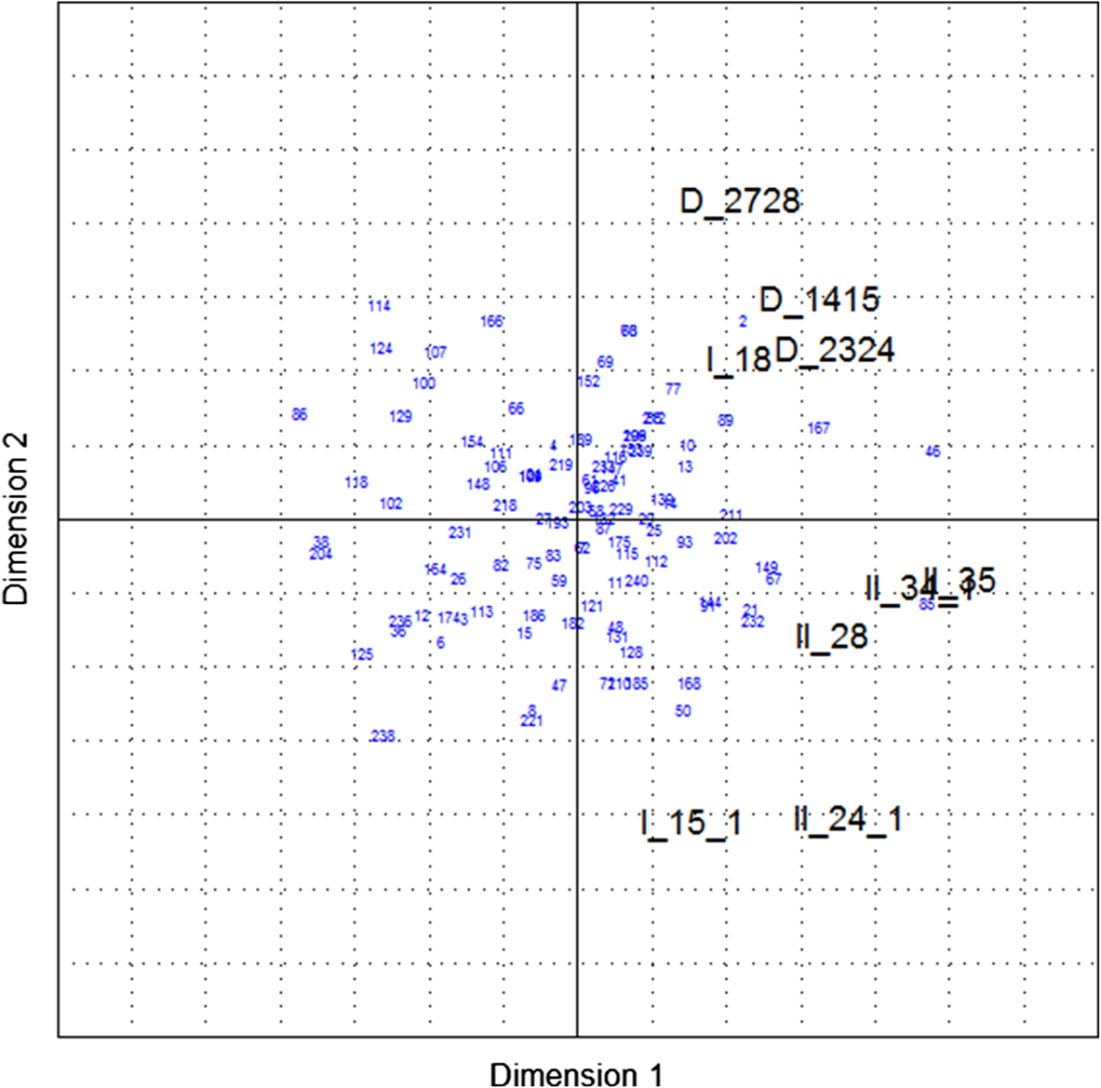
**Bi-plot showing the results of the principal component analysis of** ***Group 1*** **.** Black figures represent the dependent variables and blue figures the herds.

**Figure 2:**
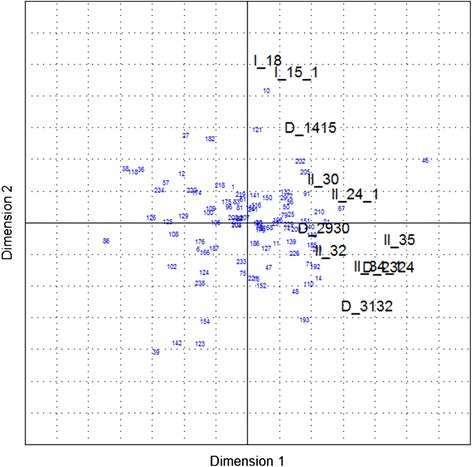
**Bi-plot showing the results of the principal component analysis of** ***Group 2*** **.** Black figures represent the dependent variables and blue figures the herds.

The results are visualized in biplots for the first two dimensions, explaining approximately 47% of the data variability for *Group 1* (Figure 1) and approximately 42% for *Group 2* (Figure 2). The plots show the correlation structure of the responses, in which several groups of two or more correlated responses can be identified.

Some correlation between the dependent variables was observed, e.g. between II_34.1 (‘*How would you estimate the efficacy of the vaccine?*’) and II_35 (‘*How is the following sentence matching your opinion: ‘My positive expectations of the vaccine’s effect in the pig herd were fulfilled.*’), but this could be confirmed only for *Group 1* and not for *Group 2*. In contrast, the variables D_2930 (‘*Change in the uniformity of growth in the fattening unit due to the use of a PCV2 vaccine*’ (farmers’ suggestion)’) and I_15.1 (‘*How would you estimate the overall health status of the pig herd after starting to use the PCV2 vaccine (i.e. today!)?*’) do not seem to be correlated. Conclusively, the results of the principal component analysis did not lead to a further reduction of dependent variables or selection of a representative dependent variable, because of the correlation structure amongst them.

c) Cluster analysis

The clustering procedure was done for the same response as for the principal component analysis via the SAS® procedure PROC FASTCLUS (SAS/STAT® (1999) - *User’s Guide Version 8*, SAS® Institute Inc – Cary NC) using the k-means method. The applied procedure resulted in two distinct clusters for each group:

- GREEN cluster with n =60 for *Group 1* and n =64 for *Group 2*,
- RED cluster with n =46 for *Group 1* and n =35 for *Group 2*.

**Figure 3:**
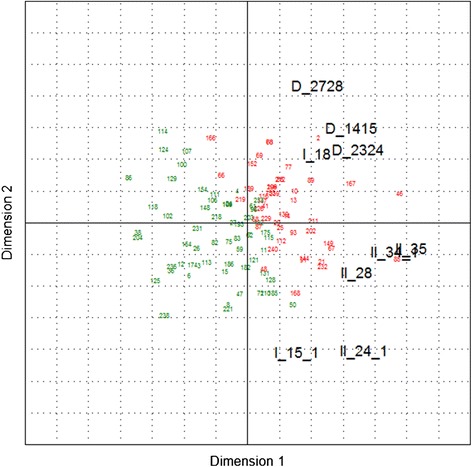
**Bi-plot showing the results of a principal component analysis including response clustering for** ***Group 1*** **.** Black figures represent the dependent variables and green/red figures the herds.

**Figure 4:**
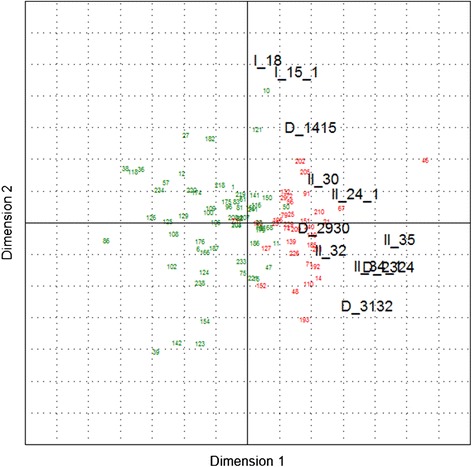
**Bi-plot showing the results of a principal component analysis including response clustering for** ***Group 2*** **.** Black figures represent the dependent variables and green/red figures the herds.

As a graphical representation of the clustering results, the GREEN and RED cluster were coloured in the PCA biplots (Figures 3 and 4) showing a quite good cluster separation in the first two dimensions (for detailed results see Additional files 1 and 2). With exception of I_15.1 (*‘How would you estimate the overall health status of the pig herd after starting to use the PCV2 vaccine (i.e. today!)?’*), on average the GREEN cluster has lower (=’better’) scores than the RED cluster and the highest differences can be seen for the responses II_34.1 (*‘How would you estimate the efficacy of the vaccine?’*) and II_35 (*‘How is the following sentence matching your opinion: ‘My positive expectations of the vaccine’s effect in the pig herd were fulfilled’*).

d) Selection of independent variables and test of association

Table 4 summarizes the p-values of the individual Fisher’s tests comparing GREEN versus RED cluster for the corresponding X-variables. Overall eight X-Variables with p-value <0.2 were selected for further modelling analysis.

**Table 4:** **p-values of the individual Fisher tests comparing green versus red cluster**

**Independent X-variable**	**p-value (Fisher test)**	
	***Group 1***	***Group 2***
Reasons for using the vaccine were either wasting, mortality or PDNS, but were not respiratory diseases, enteritis and/or marketing. (veterinarians’ answer)	0.423	0.144
Diagnosis of other diseases than PCVD	0.071	1.000
Acclimatisation of gilts	0.258	na
Vaccination of sows against PRRSV	0.395	na
Vaccination of gilts against PRRSV	0.334	na
Frequency of iron injection in piglets	0.040	na
Age of piglets when vaccinated against PCV2	0.075	na
Reasons for using the vaccine were either wasting, mortality or PDNS, but were not respiratory diseases, enteritis and/or marketing (farmers’ answer)	0.031	0.531
Overall health status of the pig herd prior to the use of the PCV2 vaccine (veterinarians’ answer)	0.001	0.123
Overall health status of the pig herd prior to the use of the PCV2 vaccine (farmers’ answer)	0.000	0.000
Time between first use of the PCV2 vaccine and the telephone interview	na	0.725
Change in the timing of PCV2 vaccination since started	na	0.058
Actual use of the PCV2 vaccine at the time of the interview (veterinarians’ answer)	na	0.343
Actual use of the PCV2 vaccine at the time of the interview (farmers’ answer)	na	0.285

na: not applicable.

e) Logistic regression model

On the basis of the cluster analyses and the selected eight X-variables two logistic regression models were used for *Group 1* and *Group 2*, respectively, with the FORWARD (SLENTRY =0.1) stepwise model selection option allowing for the inclusion of main effects and two-way interaction terms. Not considered in this selection were the variables II_23 (*‘How would you estimate the overall health status of the pig herd prior to the use of the PCV2 vaccine?’ farmer’s answer*) and I_14 (*‘How would you estimate the overall health status of the pig herd prior to the use of the PCV2 vaccine?’ veterinarian’s answer*). The procedure led to the following X variables in a final model

- *Group 1: ‘Diagnosis of other diseases than PCVD’, ‘Frequency of iron injection in piglets’, ‘Reasons for using the vaccine’, combination of ‘Diagnosis of other diseases than PCVD’ and ‘Reasons for using the vaccine’*
- *Group 2: ‘Actual use of the PCV2 vaccine at the time of the interview’, ‘Time of vaccinating piglets not changed or changed to another point in piglets’ life’*

Overall, only a few variables could be identified as influencing veterinarians’ and farmers’ ‘satisfaction’ with the PCV2 vaccine. These variables were not identical in *Group 1* and *Group 2* (Tables 5 and 6). This indicates that satisfaction among piglet producers, owners of only fattening pigs and farmers operating a 1-site production system may be influenced by different criteria.

**Table 5:** **Odds ratio estimates and 95% confidence intervals for the ‘GREEN cluster’ in the final model for** ***Group 1***

**Independent X-variable**	**OR**	**95% CI**
Only one application of iron in suckling pigs	**2.83**	1.18-6.80
No findings of other diseases than PCVD, when reasons for using the vaccine were respiratory disease, enteritis and/or marketing	**0.16**	0.05-0.56
No findings of other diseases than PCVD, when reasons for using the vaccine were wasting, mortality and/or PDNS, but not respiratory disease, enteritis and/or marketing	1.32	0.35-5.05
Reasons for using the vaccine were neither wasting nor mortality nor PDNS, but were respiratory diseases, enteritis and/or marketing. Simultaneously, no findings of other diseases in the herd	**0.10**	0.02-0.45
Reasons for using the vaccine were neither wasting nor mortality nor PDNS, but were respiratory diseases, enteritis and/or marketing. Simultaneously, findings of other diseases in the herd	0.84	0.29-2.41

Numbers in bold font indicate that the odds ratios are significantly different from one at the 5-percent level of significance.

**Table 6:** **Odds ratio estimates and 95% confidence intervals for the ‘GREEN cluster’ identified in the final model for** ***Group 2***

**Independent X-variable**	**OR**	**95% CI**
Time of vaccinating piglets not changed or change to a later point in piglets’ life instead of earlier point in time	**10.29**	1.56-67.9
Time of vaccinating piglets not changed instead of changing to an earlier point in piglets’ life	**5.00**	1.18-21.1
PCV2 vaccine still in use instead of no longer in use	5.02	0.67-37.6

Numbers in bold font indicate that the odds ratios are significantly different from one at the 5-percent level of significance.

Tables 5 and 6 show the p-values and odds ratio estimates for the final models including 95% confidence intervals. The odds ratio estimates had been calculated with the ODDSRATIO option of the SAS® procedure PROC LOGISTIC available only in Version 9.2.

## Discussion

The primary aim of the present study was to evaluate the reliability of operational data from pig herds and performance ratings by veterinarians and farmers collected during telephone interviews for the assessment of the ‘safety’ and ‘efficacy’ of a specific PCV2 vaccination in piglets. To the best knowledge of the authors, the feasibility of a telephone survey as an instrument for examining different drivers of using a vaccine in livestock, and as for examining its safety and efficacy in a large number of herds has not been described before.

Telephone interviews with customers, usually aimed to gather information about satisfaction with products or to explore the expectations of clients, are a frequently applied technique in the context of market research [10]. In contrast, veterinary research, especially in livestock animals, is mainly linked with classical on-farm interviews followed by an examination of the animals, the environment and the management. This procedure is currently ranked as the ‘gold standard’ and scientific reports based exclusively on interviews by telephone are rarely published. Interestingly, data obtained during a face to face interview can be of lower quality than such data collected via a telephone interview, because in the anonymous situation of a telephone call, the interviewees are more likely to give answers reflecting the reality [8]. Moreover, the latter technique is more cost effective than on-site visits and, if a considerable number of cases is investigated, is more time efficient [6]. The present study was to be performed under economic constraints as well as during a short time period. Therefore, a telephone survey was considered as the method of choice.

The response rate of about 80%, achieved in the present study, was considered to be highly satisfactory. Analysing the reasons for non-responding, it can be assumed that no bias was introduced by those veterinarians and farmers who had refused their participation. Other studies applying telephone interviews as the investigational tool have reported significantly lower response rates, but were still seen as relevant [8,11].

Measures of the safety and efficacy of the PCV2 vaccine involved were extrapolated from parameters assessing farmers’ and veterinarians’ overall ‘satisfaction’ with the product. This approach was chosen, because the comparison of this subjective data is more reliable than using production data (e.g. weight gain, feed consumption, etc.), if these data have not been determined under standardized conditions [12]. Marking with a nominal scale from 1 (excellent) to 6 (unsatisfactory) usually represents accurately the personal experience with a product. Calculating the increase or decrease after the introduction of any procedure allows for a comparison of relative values describing the effect of this procedure on the corresponding parameter. It is noteworthy that the relative values can be biased by professional marketing activities as has been shown for pharmaceuticals in human medicine [13], but this would usually affect only the magnitude and not the trend of the figures.

In order to determine the ‘satisfaction’ (‘dependent variable’) and to explore related parameters (‘independent variables’), two different statistical methods were tested for their suitability to separate different multivariate outcomes. The principal components analysis did not lead to a further reduction of the dependent variables or the identification of representative dependent variables. The cluster analysis resulted in a sufficient separation of highly and less satisfied cases. Further tests for association and modelling approaches were conducted using the two clusters, which had been identified with this descriptive method. In this specific study the principal component analysis was not able to reduce the dependent variables or identify representative dependent variables due to the almost unanimous positive response when asked for the satisfaction with the PCV2 vaccination. However, for a similar type of study, the same set of statistical methods would be applied in the same order and a principal component analysis might in this case result in a reduction of the dependent variables, which would be used as input for a subsequent cluster analysis and/or modelling approach.

Interestingly, only very few of the parameters listed in Tables 2 and 3 were associated with the overall satisfaction with the vaccine. Finally, three parameters for *Group 1* and two for *Group 2* were identified as ‘risk factors’ influencing the overall ‘satisfaction’ with the vaccine (Table 5 and 6). Farmers and veterinarians were less satisfied, when reasons for using the vaccine were not PCVD, wasting and/or mortality, but any other disease in the farm, which had not been properly diagnosed. These findings are conclusive since any effect of the vaccine in the absence of PCVD, or other diseases associated with PCV2 infection [14], is unlikely. Hence, these biologically sound results underline the suitability of the methods used in this study. The apparently protective effect of applying iron to piglets only once instead of twice cannot be explained. In humans, iron overload is associated with reduced phagocytosis by macrophages and monocytes [15] and a decrease of the efficiency of the unspecific immune system [16]. Whether these effects also occur in pigs and could impact the efficacy of any vaccination remains speculative. In *Group 2* the ‘satisfaction’ with the vaccine was associated with the time when the pigs were vaccinated. Taking the large confidence intervals into account, the results should be interpreted with caution, if at all [17].

Overall there was very little influence on the generally excellent satisfaction rate found in this study, which is in line with the PCV2 vaccine evaluated in this study having the highest global market shares as shown by market research figures [18]. The overall approach used in this study was feasible to scientifically assess ‘satisfaction’, and to determine factors influencing farmers’ and veterinarians’ opinion about ‘safety’, ‘efficacy’ and handling of a new vaccine. Since no strong associations were found between ‘dissatisfaction of users’ and the various parameters assessed in this study, it can be assumed that vaccine’s safety profile is high and that potential factors leading to a decreased efficacy of the product do not play any role under field conditions.

## Conclusions

It was demonstrated that a telephone survey using standardised questionnaires may be an efficient method to investigate safety and efficacy aspects of vaccines as well as customer satisfaction on a broad basis. However, due to the unexpected almost unanimous positive response to the vaccine, the present study does not allow a conclusive evaluation of the use of telephone surveys in general. Nonetheless, this study describes a suitable example of how data from field trials including a large number of herds could be assessed and analysed.

## Additional files

Additional file 1: **Supplementary material describing the individual and cumulated variance explained in each dimension and the biplots coordinates for the observations and variables for the first 4 dimensions of the principal component analysis.**

Additional file 2: **Standardised questionnaire (in German) used to better understand the factors influencing the performance of a vaccine in a large number of herds and to estimate customers’ satisfaction (i.e. safety and efficacy of the vaccine) based on data assessed with the help of a telephone poll.**
