# Supplementary material for: Reliability of operational data from pig herds and performance ratings by veterinarians and pig farmers collected during telephone interviews for the evaluation of a PCV2 piglet vaccination
Source: BMC Vet Res. 2014 Oct 28;10:260. doi: 10.1186/s12917-014-0260-1 (PMC4213548; doi:10.1186/s12917-014-0260-1)
Supplement: Supplementary file 1 — Supplementary material describing the individual and cumulated variance explained in each dimension and the biplots coordinates for the observations and variables for the first 4 dimensions of the principal component analysis. [file 12917_2014_260_MOESM1_ESM.docx]

The following tables show for the performed PCAs

- the % of Variance explained in each dimension and cumulated
- the biplots coordinates for the observations and variables for the first 4 dimensions

The “importance” of each variable as well as their dependencies on each other for the corresponding dimension can also be seen in the PCA biplots.

More technical details on the applied PCA procedure and settings of the %BIPLOT macro can be found in Friendly M: **System for Statistical Graphics.** Carey, NC, USA: SAS Insitute; 1991.

Ingelvac CircoFLEX §17c

PCA Responses

______________________________________________________________________________

Standardization Type: STD (VARDEF = N - 1 )

Singular values and variance accounted for

Singular Values Percent Cum % Histogram of %

17.3474 31.84 31.84 ****************************************

11.9359 15.08 46.92 *******************

11.2504 13.39 60.31 *****************

9.9610 10.50 70.81 *************

9.3561 9.26 80.08 ************

8.0720 6.89 86.97 *********

7.8066 6.45 93.42 ********

6.4131 4.35 97.77 *****

4.5875 2.23 100.00 ***

OBS / VARS ratio: 0.468655 Scale: 1

Biplot Factor Type

Symmetric

Biplot coordinates

______________________________________________________________________________

StatAna Ingelvac CircoFLEX §17c PCA Responses.sas

Ingelvac CircoFLEX §17c

PCA Responses

______________________________________________________________________________

DIM1 DIM2 DIM3 DIM4

OBS 10 0.3745 0.2656 0.9735 -0.0765

OBS 100 -0.5162 0.4700 0.3530 0.1550

OBS 102 -0.6246 0.0692 -0.1818 -0.1363

OBS 104 -0.1543 0.1590 0.0120 0.2175

OBS 106 -0.2722 0.1925 0.0720 0.0472

OBS 107 -0.4745 0.5790 0.1462 0.3193

OBS 109 -0.1543 0.1590 0.0120 0.2175

OBS 11 0.1310 -0.2044 -0.4312 0.2272

OBS 110 0.1475 -0.5409 -0.7148 0.2619

OBS 111 -0.2533 0.2387 0.3071 0.0396

OBS 112 0.2657 -0.1311 0.1388 -0.5018

OBS 113 -0.3162 -0.3021 0.4681 -0.3418

OBS 114 -0.6632 0.7359 -0.4938 0.0836

OBS 115 0.1702 -0.1068 0.0439 0.3724

OBS 116 0.1281 0.2256 0.0747 0.0356

OBS 118 -0.7412 0.1358 0.4967 0.1393

OBS 12 -0.5199 -0.3116 0.3297 0.4397

OBS 121 0.0548 -0.2818 0.8054 -0.0821

OBS 124 -0.6591 0.5887 0.0845 -0.4776

OBS 125 -0.7248 -0.4451 0.0476 0.2156

OBS 128 0.1816 -0.4349 0.3982 -0.2525

OBS 129 -0.5940 0.3622 0.2386 0.2174

OBS 13 0.3679 0.1886 -0.0831 0.3903

OBS 131 0.1391 -0.3869 0.1311 0.5437

OBS 132 0.0901 0.0164 0.4633 -0.3014

OBS 139 0.2901 0.0808 -0.1975 0.4526

OBS 14 0.3138 0.0655 -0.5140 -0.2484

OBS 144 0.4503 -0.2674 -0.0049 0.5374

______________________________________________________________________________

StatAna Ingelvac CircoFLEX §17c PCA Responses.sas

Ingelvac CircoFLEX §17c

PCA Responses

______________________________________________________________________________

DIM1 DIM2 DIM3 DIM4

OBS 147 0.1194 0.1872 -0.0702 0.0238

OBS 148 -0.3311 0.1309 0.1927 0.1019

OBS 149 0.6375 -0.1484 -0.3241 0.2797

OBS 15 -0.1738 -0.3706 -0.3493 -0.3361

OBS 152 0.0394 0.4818 -0.4147 -0.1625

OBS 153 0.1812 0.2493 -0.0976 -0.0425

OBS 154 -0.3517 0.2774 -0.7730 -0.0299

OBS 164 -0.4756 -0.1561 0.1321 -0.0242

OBS 166 -0.2829 0.6807 -0.0992 0.0294

OBS 167 0.8166 0.3213 0.4390 -0.2556

OBS 168 0.3781 -0.5422 -0.5023 0.4716

OBS 174 -0.4298 -0.3227 0.2175 0.0258

OBS 175 0.1427 -0.0672 -0.1323 0.6108

OBS 182 -0.0101 -0.3376 0.4418 -0.1443

OBS 185 0.2028 -0.5458 -0.1651 -0.3771

OBS 186 -0.1446 -0.3166 -0.0385 -0.2042

OBS 189 0.0118 0.2851 0.2599 -0.1507

OBS 193 -0.0617 0.0025 -0.6668 -0.6634

OBS 196 0.1972 0.2950 0.0442 -0.0385

OBS 2 0.5601 0.6796 -0.6301 -0.0474

OBS 202 0.4994 -0.0517 0.1129 1.0731

OBS 203 0.0164 0.0526 -0.1153 0.6464

OBS 204 -0.8559 -0.1010 -0.2625 0.0301

OBS 209 0.1972 0.2950 0.0442 -0.0385

OBS 21 0.5872 -0.2958 -0.2623 0.1037

OBS 211 0.5242 0.0275 -0.3151 0.3039

OBS 212 0.2590 0.3570 0.0168 -0.1049

OBS 218 -0.2369 0.0622 0.3016 0.3496

______________________________________________________________________________

StatAna Ingelvac CircoFLEX §17c PCA Responses.sas

Ingelvac CircoFLEX §17c

PCA Responses

______________________________________________________________________________

DIM1 DIM2 DIM3 DIM4

OBS 219 -0.0486 0.1975 0.2555 -0.0800

OBS 221 -0.1486 -0.6687 -0.4879 -0.2896

OBS 226 0.0930 0.1271 -0.2273 0.0282

OBS 229 0.1523 0.0448 0.1477 -0.4777

OBS 231 -0.3874 -0.0339 0.2618 -0.0949

OBS 232 0.5925 -0.3301 -0.0386 0.4539

OBS 233 0.0902 0.1878 -0.3006 0.1861

OBS 236 -0.5930 -0.3323 -0.3084 -0.0854

OBS 238 -0.6505 -0.7186 -0.4365 0.0462

OBS 239 0.2164 0.2439 0.2083 -0.2703

OBS 24 -0.1451 0.1678 0.1098 0.2325

OBS 240 0.2039 -0.1932 0.1661 -0.4355

OBS 25 0.2602 -0.0284 0.0970 -0.2387

OBS 26 -0.3933 -0.1914 -0.3084 -0.2094

OBS 27 -0.1081 0.0158 0.3625 0.3482

OBS 29 0.2328 0.0113 -0.0792 -0.0004

OBS 3 -0.3785 -0.3295 -0.2976 -0.2165

OBS 36 -0.5986 -0.3688 0.4054 -0.1598

OBS 38 -0.8606 -0.0617 0.3705 0.8688

OBS 4 -0.0800 0.2650 -0.3224 -0.2644

OBS 41 0.1468 0.1475 0.1060 -0.2146

OBS 46 1.1961 0.2430 0.2057 0.0125

OBS 47 -0.0604 -0.5465 -0.3582 -0.3603

OBS 48 0.1293 -0.3545 -0.4975 -0.5589

OBS 50 0.3570 -0.6356 -0.1297 0.4274

OBS 55 0.2590 0.3570 0.0168 -0.1049

OBS 58 0.0690 0.0398 -0.0084 -0.1522

OBS 59 -0.0564 -0.1944 0.0912 -0.2749

______________________________________________________________________________

StatAna Ingelvac CircoFLEX §17c PCA Responses.sas

Ingelvac CircoFLEX §17c

PCA Responses

______________________________________________________________________________

DIM1 DIM2 DIM3 DIM4

OBS 6 -0.4568 -0.4081 -0.2630 -0.1324

OBS 61 0.0498 0.1470 0.1094 0.1196

OBS 62 0.0214 -0.0866 0.2056 -0.3372

OBS 66 -0.2029 0.3861 0.2453 0.2156

OBS 67 0.6650 -0.1880 -0.1479 0.0414

OBS 68 0.1790 0.6516 -0.3277 -0.2911

OBS 69 0.1012 0.5439 -0.4421 -0.2288

OBS 7 0.0214 -0.0866 0.2056 -0.3372

OBS 70 0.1790 0.6516 -0.3277 -0.2911

OBS 72 0.1038 -0.5426 0.2838 -0.1901

OBS 75 -0.1454 -0.1370 -0.4455 0.1207

OBS 77 0.3286 0.4534 0.1192 -0.1606

OBS 8 -0.1518 -0.6322 0.4057 -0.3050

OBS 82 -0.2518 -0.1444 0.2407 -0.1404

OBS 83 -0.0784 -0.1082 0.0568 0.0059

OBS 85 1.1769 -0.2722 0.4292 -0.0026

OBS 86 -0.9309 0.3652 -0.1610 0.2828

OBS 87 0.0905 -0.0173 0.1751 -0.4114

OBS 89 0.4993 0.3471 -0.0080 0.2682

OBS 91 0.4445 -0.2837 0.0352 0.0340

OBS 93 0.3648 -0.0662 0.6872 -0.3976

OBS 96 0.0503 0.1178 -0.0397 0.0979

VAR I_15_1 0.6264 -1.5879 1.7501 0.8377

VAR D_1415 1.3030 1.2420 1.2427 -0.5995

VAR I_18 0.8803 0.9089 2.1418 0.2635

VAR II_24_1 1.4696 -1.5687 -0.0751 -0.1799

VAR D_2324 1.3924 0.9620 -0.3994 -0.8579

VAR II_28 1.3766 -0.5901 -0.6653 1.9854

______________________________________________________________________________

StatAna Ingelvac CircoFLEX §17c PCA Responses.sas

Ingelvac CircoFLEX §17c

PCA Responses

______________________________________________________________________________

DIM1 DIM2 DIM3 DIM4

VAR D_2728 0.8778 1.7673 -0.6983 1.7610

VAR II_34_1 1.8536 -0.3263 -0.9197 -0.7327

VAR II_35 2.0694 -0.2861 -0.3386 -0.6946

______________________________________________________________________________

StatAna Ingelvac CircoFLEX §17c PCA Responses.sas

Ingelvac CircoFLEX §17c

PCA Responses

______________________________________________________________________________

Standardization Type: STD (VARDEF = N - 1 )

Singular values and variance accounted for

Singular Values Percent Cum % Histogram of %

17.6954 29.05 29.05 ****************************************

11.9052 13.15 42.20 ******************

11.6698 12.63 54.83 *****************

11.0606 11.35 66.18 ****************

9.5143 8.40 74.57 ************

8.7191 7.05 81.63 **********

7.9540 5.87 87.49 ********

7.4083 5.09 92.59 *******

5.5757 2.88 95.47 ****

5.2104 2.52 97.99 ***

4.6568 2.01 100.00 ***

OBS / VARS ratio: 0.693336 Scale: 1

Biplot Factor Type

Symmetric

Biplot coordinates

______________________________________________________________________________

StatAna Ingelvac CircoFLEX §17c PCA Responses.sas

Ingelvac CircoFLEX §17c

PCA Responses

______________________________________________________________________________

DIM1 DIM2 DIM3 DIM4

OBS 1 -0.1124 0.2957 0.1443 0.5145

OBS 10 0.1508 1.0567 -0.1502 -0.5128

OBS 100 -0.3044 0.0943 0.4129 -0.3248

OBS 102 -0.5938 -0.3305 0.4331 -0.0398

OBS 104 -0.0831 -0.0054 -0.1064 -0.0844

OBS 105 0.1071 -0.0293 -0.1672 -0.1636

OBS 106 -0.2471 0.0141 -0.1029 -0.2871

OBS 108 -0.5793 -0.0806 0.0075 -0.0491

OBS 109 -0.2858 0.1255 0.1493 -0.1789

OBS 11 0.2304 -0.1474 0.3059 0.3241

OBS 110 0.4797 -0.4719 -0.0242 0.7704

OBS 116 0.0754 0.1511 -0.1285 -0.2495

OBS 118 -0.8949 0.4108 0.3402 -0.3220

OBS 12 -0.5161 0.4020 -0.3368 0.3966

OBS 121 0.0811 0.7482 -0.2298 -0.0744

OBS 123 -0.3685 -0.9450 -0.0533 0.3717

OBS 124 -0.3346 -0.3760 -0.4404 -0.5123

OBS 125 -0.6269 0.0237 -0.2724 0.5459

OBS 126 -0.7511 0.0517 0.9797 0.8020

OBS 127 0.1496 -0.1870 -0.2179 -0.0453

OBS 129 -0.4999 0.0688 0.0866 -0.2175

OBS 132 0.2976 0.2540 -0.3552 -0.2307

OBS 139 0.3430 -0.1395 0.1144 -0.1659

OBS 14 0.5623 -0.4273 0.2174 -0.1060

OBS 141 0.0613 0.2291 0.4115 0.1781

OBS 142 -0.5500 -0.9363 -0.1688 -1.3818

OBS 149 0.5105 -0.0817 -0.3059 -0.1478

OBS 15 0.0738 -0.4291 -0.2168 -0.0341

______________________________________________________________________________

StatAna Ingelvac CircoFLEX §17c PCA Responses.sas

Ingelvac CircoFLEX §17c

PCA Responses

______________________________________________________________________________

DIM1 DIM2 DIM3 DIM4

OBS 150 0.1598 0.2127 -0.3218 -0.1130

OBS 151 0.4552 0.0302 0.5475 0.3261

OBS 152 0.1099 -0.4826 0.1244 -0.3049

OBS 154 -0.3330 -0.7643 0.3941 -0.2028

OBS 16 0.1071 -0.0293 -0.1672 -0.1636

OBS 160 0.0250 0.1294 0.1875 -0.4777

OBS 166 -0.3302 -0.2242 0.6376 -0.4873

OBS 168 0.1632 -0.0232 -0.5661 0.2761

OBS 174 -0.3972 0.2517 -0.0105 0.3044

OBS 175 -0.1605 0.1783 -0.2626 0.0412

OBS 176 -0.3729 -0.1385 -0.3359 0.4176

OBS 179 0.1037 -0.0429 -0.4022 0.0751

OBS 182 -0.2896 0.6789 -0.1018 -0.1348

OBS 185 0.5093 -0.1646 0.0191 0.2929

OBS 186 0.0568 -0.1473 -0.2453 0.0095

OBS 187 -0.2563 -0.1906 -0.1834 0.1610

OBS 192 0.5344 -0.3337 0.5937 0.2431

OBS 193 0.4497 -0.7521 0.7733 0.1807

OBS 195 0.0868 0.0076 0.7711 -0.5723

OBS 196 0.2443 0.0352 -0.0709 -0.3711

OBS 20 0.0936 0.0166 -0.4401 0.2692

OBS 202 0.4188 0.4997 0.2827 0.3238

OBS 203 -0.0831 -0.0054 -0.1064 -0.0844

OBS 205 0.4595 0.4160 0.4343 0.2788

OBS 207 -0.0214 0.0541 0.2693 0.5997

OBS 208 -0.1129 0.0536 0.3971 0.5854

OBS 209 0.3874 -0.0354 0.1833 -0.3186

OBS 21 0.6238 0.0219 -0.0492 0.1840

______________________________________________________________________________

StatAna Ingelvac CircoFLEX §17c PCA Responses.sas

Ingelvac CircoFLEX §17c

PCA Responses

______________________________________________________________________________

DIM1 DIM2 DIM3 DIM4

OBS 210 0.5707 0.1022 0.2271 -0.1585

OBS 211 0.5523 -0.1978 0.0218 -0.2430

OBS 218 -0.2124 0.3092 -0.2419 0.1865

OBS 219 -0.0511 0.2395 0.5098 -0.3009

OBS 220 -0.4329 0.2712 -0.0070 -0.0507

OBS 221 0.0485 -0.4253 -0.3985 0.3536

OBS 226 0.3745 -0.2268 0.2373 0.1184

OBS 229 0.3273 -0.0002 -0.0463 -0.4202

OBS 23 0.2197 0.0066 -0.0714 -0.3761

OBS 231 -0.0831 0.0284 0.2459 0.0256

OBS 233 -0.0436 -0.2940 0.1347 -0.3407

OBS 234 -0.6872 0.2725 0.4103 -0.4695

OBS 238 -0.3676 -0.4619 -0.3302 0.6852

OBS 24 -0.0605 0.0465 -0.0696 -0.1111

OBS 240 0.4877 -0.0195 -0.2846 -0.1888

OBS 241 0.0464 0.1197 -0.1295 -0.2542

OBS 25 0.3455 0.0807 -0.1443 -0.2234

OBS 27 -0.4799 0.7028 -0.0410 -0.0556

OBS 29 0.2866 0.2087 0.5265 0.2789

OBS 36 -0.8296 0.4351 -0.1154 -0.0283

OBS 38 -0.9541 0.4373 -0.2897 0.3007

OBS 39 -0.7166 -1.0099 -0.1989 0.3742

OBS 46 1.3986 0.5033 0.5425 0.1188

OBS 47 0.1729 -0.3393 -0.4876 0.2749

OBS 48 0.3759 -0.5270 -0.2820 -0.1660

OBS 50 0.3066 0.1402 -0.7277 0.3384

OBS 56 0.3398 0.1761 0.3856 0.1278

OBS 57 -0.6398 0.3298 -0.1401 0.2447

______________________________________________________________________________

StatAna Ingelvac CircoFLEX §17c PCA Responses.sas

Ingelvac CircoFLEX §17c

PCA Responses

______________________________________________________________________________

DIM1 DIM2 DIM3 DIM4

OBS 6 -0.3841 -0.1976 -0.2514 0.2499

OBS 61 -0.0297 0.1960 -0.1596 -0.1873

OBS 67 0.7431 0.1237 0.2470 0.1436

OBS 7 -0.0511 0.2057 0.1574 -0.4108

OBS 71 0.4873 -0.3157 -0.5035 -0.0658

OBS 72 0.3350 0.2262 -0.5625 0.1291

OBS 74 0.3124 -0.0330 -0.0440 -0.4309

OBS 75 -0.0314 -0.3842 -0.2480 0.0280

OBS 79 0.2954 0.0719 0.3167 0.2805

OBS 81 -0.0553 0.1334 -0.2315 -0.0210

OBS 82 -0.0605 0.0465 -0.0696 -0.1111

OBS 83 -0.0855 0.1760 -0.0353 0.1535

OBS 86 -1.1044 -0.1276 0.6453 0.1172

OBS 91 0.4682 0.2442 -0.6852 0.1189

OBS 96 -0.1403 0.1361 -0.4891 -0.1483

VAR I_15_1 0.6299 1.9481 -1.2195 0.3783

VAR D_1415 0.7897 1.2452 0.8075 -1.6969

VAR I_18 0.2596 2.0944 0.2547 -0.6303

VAR II_24_1 1.3659 0.4133 -0.6539 1.2466

VAR D_2324 1.7732 -0.5094 0.3462 -0.6549

VAR II_30 0.9653 0.5937 1.4419 1.7665

VAR D_2930 0.9573 -0.0201 1.9147 0.8967

VAR II_32 1.0523 -0.3055 -1.5676 0.8387

VAR D_3132 1.4983 -1.0099 0.3907 -0.8223

VAR II_34_1 1.6451 -0.5003 -0.6317 -0.3221

VAR II_35 1.9211 -0.1622 -0.4048 -0.5000

______________________________________________________________________________

StatAna Ingelvac CircoFLEX §17c PCA Responses.sas
