# Supplementary material for: Reliability of operational data from pig herds and performance ratings by veterinarians and pig farmers collected during telephone interviews for the evaluation of a PCV2 piglet vaccination
Source: BMC Vet Res. 2014 Oct 28;10:260. doi: 10.1186/s12917-014-0260-1 (PMC4213548; doi:10.1186/s12917-014-0260-1)
Supplement: Supplementary file 2 — Standardised questionnaire (in German) used to better understand the factors influencing the performance of a vaccine in a large number of herds and to estimate customers’ satisfaction (i.e. safety and efficacy of the vaccine) based on data assessed with the help of a telephone poll. [file 12917_2014_260_MOESM2_ESM.docx]

**Ingelvac^®^ CircoFLEX - Screening**

| **Tierarzt**        Name, Vorname |
| --- |
| Straße, HsNr. |
| PLZ, Ort |
| Telefon |
| Telefax |

| **Tierbesitzer**        Name, Vorname |
| --- |
| Straße, HsNr. |
| PLZ, Ort |
| Telefon |
| Telefax |

Grüne Markierung: Fragen nicht bei reinen Mästern (nur Kombi/ Ferkelerzeuger)

Gelbe Markierung: Fragen nicht bei reinen Ferkelerzeugern (nur Kombi/ Mast)

**Befragung des Hoftierarztes**

**Erklären Sie sich bereit mit den von Ihnen betreuten Betrieben, die im Rahmen der Ausnahmegenehmigung nach §17c Ingelvac® CircoFLEX geimpft haben, an dieser Studie teilzunehmen?**

nein  ja

**Produktionstyp dieses Bestandes**

Kombibestand

Ferkelerzeugerbestand bis 8 kg

Ferkelerzeugerbestand bis 28 kg

Ferkelaufzucht 8 bis 28 kg

Mastbestand (28-110 kg)

**Aus welchem Grund wird/ wurde Ingelvac® CircoFLEX eingesetzt**

**Kümmern**

bei  Saugferkeln  Absetzferkeln  Vormast  Mittel-/ Endmast

**Atemwegserkrankungen**

bei  Saugferkeln  Absetzferkeln  Vormast  Mittel-/ Endmast

**Durchfallerkrankungen**

bei  Saugferkeln  Absetzferkeln  Vormast  Mittel-/ Endmast

**Hautveränderungen (PDNS)**

bei  Saugferkeln  Absetzferkeln  Vormast  Mittel-/ Endmast

**Erhöhte Verluste**

bei  Saugferkeln  Absetzferkeln  Vormast  Mittel-/ Endmast

**Erhöhung der Vermarktungsfähigkeit**

bei  Saugferkeln  Absetzferkeln  Vormast  Mittel-/ Endmast

**andere Gründe**:______________________________________________

______________________________________________________________

bei  Saugferkeln  Absetzferkeln  Läufern  Mastschweinen

**Wurden Sektionen/ Laboruntersuchungen zur PCV2-Diagnostik vorab durchgeführt**

nein

ja

Sektionen  Histologie

wann: ________________

wie viele Tiere: _________

Ergebnis: ___________________________________________________

___________________________________________________________

___________________________________________________________

___________________________________________________________

Antigennachweis mit PCR

wann: ________________

wie viele Tiere: _________

Probenmaterial: ______________________________________________

Ergebnis: ___________________________________________________

___________________________________________________________

___________________________________________________________

___________________________________________________________

Antikörpernachweis mit ELISA

wann: ________________ und ___________________

wie viele Tiere: _________ (jüngste U.) ______________(ältere U.)

Ergebnis: ___________________________________________________

___________________________________________________________

___________________________________________________________

___________________________________________________________

**Sind Untersuchungen auf andere Erkrankungen durchgeführt worden?**

nein

ja

wann: ________________ und ___________________

wie viele Tiere: _________ (jüngste U.) ______________(ältere U.)

Methode  Sektion, Ergebnis _________________________________

___________________________________________________________

Histologie, Ergebnis _______________________________

___________________________________________________________

PCR, Ergebnis ___________________________________

___________________________________________________________

Bakteriologie, Ergebnis _____________________________

___________________________________________________________

Serologie, Ergebnis________________________________

___________________________________________________________

**Jungsauen - Eingliederung/ Quarantäne**

nein

ja

Dauer___________ (Wochen)

Separates Gebäude

Separates Abteil

**Kontakt zu Altsauen während der Eingliederung**

nein

ja

**Jungsauen - Impfprogramm**

| **Art der Impfung** | **Impfstoff** | **Impfzeitpunkt** | **Impfintervall** |
| --- | --- | --- | --- |
| **PRRS** |  |  |  |
| **Influenza** |  |  |  |
| **Rotlauf** |  |  |  |
| **Parvovirose** |  |  |  |
| **Rhinitis atrophicans** |  |  |  |
| **Colidiarrhoe** |  |  |  |
| **stallspezif. Impfstoffe** |  |  |  |
| **andere** |  |  |  |

**Altsauen – Impfprogramm**

| **Art der Impfung** | **Impfstoff** | **Impfzeitpunkt** | **Impfintervall** |
| --- | --- | --- | --- |
| **PRRS** |  |  |  |
| **PCV2** |  |  |  |
| **Influenza** |  |  |  |
| **Rotlauf** |  |  |  |
| **Parvovirose** |  |  |  |
| **Rhinitis atrophicans** |  |  |  |
| **Colidiarrhoe** |  |  |  |
| **stallspezif. Impfstoffe** |  |  |  |
| **andere** |  |  |  |

**Saug- und Absetzferkel – Impfprogramm**

| **Art der Impfung** | **Impfstoff** | **Impfzeitpunkt** | **Zeitpunkt Wdh.** |
| --- | --- | --- | --- |
| **Mycoplasma hyopneumoniae** |  |  |  |
| **PRRS** |  |  |  |
| **APP** |  |  |  |
| **PIA** |  |  |  |
| **stallspez.**  **Impfstoffe** |  |  |  |

**Wie alt sind die Tiere bei Impfung mit Ingelvac® CircoFLEX (Alter/ Produktionsstadium)**

_________ (Tage)

Produktionsstadium:

Abferkelstall

Flatdeck

Maststall

**Wurde der Impfzeitpunkt seit Einsatzbeginn schon verändert?**

nein  ja

**Wenn ja, von welchem zu welchem neuen Zeitpunkt?**

______________________________________________________________

**Wurden neben Ingelvac® CircoFLEX auch andere Impfstoffe gegen PCV2 eingesetzt?**

nein  ja

**Wenn ja, wann/ in welchem Zeitraum?**

_______________________________________________________________

**Werden um den Zeitpunkt der Impfung mit Ingelvac® CircoFLEX auch Antibiotika verabreicht?**

nein, Antibiotika werden 5 Tage vor/ nach Impfung nicht eingesetzt

manchmal

ja, innerhalb von 5 Tagen vor/ nach Impfung werden Antibiotika eingesetzt

über Futter/Wasser

über Spritze

**Wie würden Sie die Tiergesundheit in Ihrem Bestand vor dem Einsatz von Ingelvac® CircoFLEX auf einer Schulnotenskala von 1 bis 6 beurteilen?**

1  4

2  5

3  6

**Beurteilen Sie nun die Tiergesundheit in Ihrem Bestand heute**

**(unter Einsatz von Ingelvac® CircoFLEX) auf einer Schulnotenskala von 1 bis 6**

1  4

2  5

3  6

An welchen Anzeichen/Merkmalen/Kriterien wurde eine Veränderung bemerkt:

___________________________________________________________________

___________________________________________________________________

___________________________________________________________________

___________________________________________________________________

Welche Probleme gibt es derzeit im Bestand (betroffene Altersgruppe?):

___________________________________________________________________

___________________________________________________________________

___________________________________________________________________

___________________________________________________________________

**Die Aussage:**

**„Die PCV2 assoziierten Krankheitsprobleme in diesem Bestand haben sich seit Anwendung von Ingelvac® CircoFLEX verringert?“**

trifft zu

trifft nicht zu

Bitte ziehen Sie ein Resümee:

Die Feststellung

„**Meine positiven Erwartungen an den Einsatz des Impfstoffes in diesem Bestand haben sich erfüllt.“**

trifft für mich, bewertet auf einer Schulnotenskala (1=voll; 6= gar nicht), zu.

1  4

2  5

3  6

**Bemerkungen / Erfahrungen/ offene Fragen des Tierarztes bei der Anwendung/ subjektive Einschätzung des Impferfolges im Bestand**

___________________________________________________________________

___________________________________________________________________

___________________________________________________________________

___________________________________________________________________

**Befragung des Tierhalters**

**Tierbestand**

Anzahl Sauen ____________________

Absetzalter (Tage) ____________________

Anzahl FD Plätze ____________________

Anzahl Mastplätze ____________________

**Produktionstyp**

Kombibestand

Ferkelerzeugerbestand bis 8 kg

Ferkelerzeugerbestand bis 28 kg

Ferkelaufzucht 8 bis 28 kg

Mastbestand (28-110 kg)

**Sauenherde**

In welchem Jahr wurde die Sauenherde aufgebaut: _____________________

Wurde die Herde in der Zwischenzeit aufgestockt/ vergrößert (Jahr): _____________

**Abferkelrhythmus**

1-wöchig

2-wöchig

3-wöchig

4-wöchig

sonstiges _____________________

**Aufzucht- bzw. Mastbetriebe**

**Einstallrhythmus und Gruppengröße**

Alle __________ Wochen ___________ Tiere

**Aus wie vielen verschiedenen Beständen beziehen Sie insgesamt Ihre Ferkel?**

Anzahl ____________________

**Kaufen Sie die Ferkel konstant aus den gleichen Betrieben oder wechseln Sie die Herkünfte?**

konstant

wechselnd

**Stallbelegung**

Abferkelstall

R-R, abteilweise

R-R, stallweise

kontinuierlich

Flatdeck

R-R, abteilweise

R-R, stallweise

kontinuierlich

Maststall

R-R, abteilweise

R-R, stallweise

kontinuierlich

**Verbleib von Restbeständen (z.B. Kümmerern, Kranken im Abteil) bei Neueinstallung von Ferkeln/ Masttieren**

Verbleiben in ihrer Bucht

Umstallung in Krankenbucht im selben Abteil

Umstallung in separates/n Krankenabteil/-stall

Euthanasie/ Tötung

Rückstallung zu jüngeren Gruppen

sonstiges____________________________

**Datum der ersten Anwendung von Ingelvac® CircoFLEX** _____________________

**Wird Ingelvac® CircoFLEX aktuell eingesetzt?**

ja

nein, Anwendung bis: ____________

**Gibt es Schweine im Bestand, die im Moment nicht gegen Circovirus geimpft sind?**

Sauen

Mastschweine, Alter: ____________

Ferkel im Flatdeck, Alter: _________

**Warum wird/ wurde Ingelvac® CircoFLEX eingesetzt?**

(Mehrfachnennungen möglich)

**Kümmern**

bei  Saugferkeln  Absetzferkeln  Vormast  Mittel-/ Endmast

**Atemwegserkrankungen**

bei  Saugferkeln  Absetzferkeln  Vormast  Mittel-/ Endmast

**Durchfallerkrankungen**

bei  Saugferkeln  Absetzferkeln  Vormast  Mittel-/ Endmast

**Hautveränderungen (PDNS)**

bei  Saugferkeln  Absetzferkeln  Vormast  Mittel-/ Endmast

**Erhöhte Verluste**

bei  Saugferkeln  Absetzferkeln  Vormast  Mittel-/ Endmast

**Erhöhung der Vermarktungsfähigkeit**

bei  Saugferkeln  Absetzferkeln  Vormast  Mittel-/ Endmast

**andere Gründe**:___________________________________________

____________________________________________________________

bei  Saugferkeln  Absetzferkeln  Läufern  Mastschweinen

**Wie lange bestanden die Probleme bevor ingelvac® CircoFLEX eingesetzt wurde?**

< 3 Monate

3 bis 6 Monate

> 6 Monate

**Wie alt sind die Tiere bei Impfung mit Ingelvac® CircoFLEX (Alter/Produktionsstadium)?**

_________ (Tage) _________ (Wochen)

Produktionsstadium:

Abferkelstall

Flatdeck

Maststall

**Werden gleichzeitig mit Ingelvac® CircoFLEX auch noch andere Spritzen gegeben?**

nein

ja, auf der anderen Halsseite wird auch _________________ gespritzt

ja, auf der gleichen Halsseite wird ________________________ gespritzt

getrennte Spritzen  Mischspritze

keine Angabe

**Wann werden die Ferkel kastriert?**

_____________ (Lebenstag)

**Fällt die Kastration mit der Ingelvac® CircoFLEX Impfung zusammen?**

ja

nein

**Wann bekommen die Ferkel Eisen?**

_____________ (Lebenstag) und _________ (Lebenstag)

**Wie wird/wurde die Impfung mit Ingelvac® CircoFLEX von den Tieren vertragen?**

(Mehrfachnennungen möglich)

keine Auffälligkeiten

Hautveränderungen bei Einzeltieren

Hautveränderungen bei > 10% der Tiere

Mattigkeit bei Einzeltieren

Mattigkeit bei > 10% der Tiere

Futteraufnahme vermindert bei Einzeltieren

Futteraufnahme vermindert bei > 10% der Tiere

sonstige

**Wie würden Sie die Tiergesundheit in Ihrem Bestand vor dem Einsatz von Ingelvac® CircoFLEX auf einer Schulnotenskala von 1 bis 6 beurteilen?**

1  4

2  5

3  6

**Beurteilen Sie nun die Tiergesundheit in Ihrem Bestand heute (unter Einsatz von ingelvac® CircoFLEX) auf einer Schulnotenskala von 1 bis 6?**

1  4

2  5

3  6

An welchen Anzeichen/Merkmalen/Kriterien wurde eine Veränderung bemerkt:

___________________________________________________________________

___________________________________________________________________

___________________________________________________________________

Welche Probleme gibt es derzeit im Bestand (betroffene Altersgruppe?):

___________________________________________________________________

___________________________________________________________________

___________________________________________________________________

___________________________________________________________________

**Werden Produktionsdaten erfasst**

(Mehrfachnennungen möglich)

elektronischer Sauenplaner, welcher Planer:________________________

Sauendaten handschriftlich erfasst

elektronische Mastdatenerfassung, welcher Planer:__________________

Mastdaten handschriftlich erfasst

keine Erfassung von Mastdaten

**Bitte beurteilen Sie das „Auseinanderwachsen“ der Aufzuchtferkel (Flatdeck) vor dem Einsatz von Ingelvac® CircoFLEX auf einer Schulnotenskala (1 = nie, 6= sehr deutlich)**

1  4

2  5

3  6

**Bitte beurteilen Sie das „Auseinanderwachsen“ der Aufzuchtferkel (Flatdeck) heute (unter dem Einsatz von Ingelvac® CircoFLEX) auf einer Schulnotenskala (1 = nie, 6= sehr deutlich)**

1  4

2  5

3  6

**Bitte beurteilen Sie das „Auseinanderwachsen“ der Mastgruppen vor dem Einsatz von Ingelvac® CircoFLEX auf einer Schulnotenskala (1 = nie, 6= sehr deutlich)**

1  4

2  5

3  6

**Bitte beurteilen Sie das „Auseinanderwachsen“ der Mastgruppen heute (unter dem Einsatz von Ingelvac® CircoFLEX) auf einer Schulnotenskala (1 = nie, 6= sehr deutlich)**

1  4

2  5

3  6

**Wie beurteilen Sie die Handhabung von Ingelvac® CircoFLEX auf einer Schulnotenskala?**

1  4

2  5

3  6

Warum haben Sie diese Antwort gegeben

___________________________________________________________________

___________________________________________________________________

___________________________________________________________________

**Wie beurteilen Sie die Wirksamkeit von Ingelvac® CircoFLEX auf einer Schulnotenskala?**

1  4

2  5

3  6

Warum haben Sie diese Antwort gegeben

___________________________________________________________________

___________________________________________________________________

___________________________________________________________________

Bitte ziehen Sie ein Resümee:

Die Feststellung

„**Meine positiven Erwartungen an den Einsatz des Impfstoffes in meinem Bestand haben sich erfüllt.“**

trifft für mich, bewertet nach einer Schulnotenskala (1=voll; 6= gar nicht), zu.

1  4

2  5

3  6

**Wollen sie Ingelvac® CircoFLEX in der nächsten Zeit weiter einsetzen?**

ja

nein

weiß noch nicht

Warum haben Sie diese Entscheidung getroffen (Wirksamkeit, Preis, Aufwand für Impfung etc.)

___________________________________________________________________

___________________________________________________________________

___________________________________________________________________

**Erklären Sie sich - unverbindlich - dazu bereit an weiteren Untersuchungen im Rahmen dieses Forschungsprojektes teilzunehmen?**

ja

nein

vielleicht

**Besonderheiten:**

___________________________________________________________________

___________________________________________________________________

__________________________________________________________________

**Datum EDV-Eingabe:**_________________
